# Spatiotemporal drought analysis by the standardized precipitation index (SPI) and standardized precipitation evapotranspiration index (SPEI) in Sichuan Province, China

**DOI:** 10.1038/s41598-020-80527-3

**Published:** 2021-01-14

**Authors:** Changhong Liu, Cuiping Yang, Qi Yang, Jiao Wang

**Affiliations:** 1 Institute of Environment Remediation and Human Health, Southwest Forestry University, Kunming, 650224 Yunnan China; 2 College of Ecology and Environment, Southwest Forestry University, Kunming, 650224 Yunnan China; 3 Key Laboratory of Ecological Environment Evolution and Pollution Control in Mountainous and Rural Areas of Yunnan Province, Kunming, 650224 China

**Keywords:** Climate sciences, Natural hazards

## Abstract

Drought refers to a meteorological disaster that causes insufficient soil moisture and damage to crop water balance due to long-term lack of precipitation. With the increasing shortage of water resources, drought has become one of the hot issues of global concern. The standardized precipitation index (SPI) and standardized precipitation evapotranspiration index (SPEI) can effectively reflect the changes in drought characteristics of different geomorphologies in Sichuan on time and space scales, to explore the difference in drought characteristics between different physiognomy types in Sichuan Province, We calculated the SPI and SPEI values based on the data of 44 meteorological stations in Sichuan Province from 1961 to 2019 and used Mann–Kendall trend test and multivariable linear regression method (MLR) to quantify the significance of the drought characteristic trends at different time and space scales. The results as follow: (1) The SPEI drought trend in plain and hilly regions was greater than that in plateau and mountain regions on all time scales (− 0.039 year^−1^ for 1-month in hilly, − 0.035 year^−1^ for 1-month in plain, − 0.14 year^−1^ for 1-month in plateau, − 0.026 year^−1^ for 1-month in mountain) and the magnitude of trend of eastern (− 4.4 to 0.1 year^−1^) was lager than western (− 2.1 to 2.7 year^−1^), means that the drought trends transfer from northwest to east. (2) The drought intensity in the western region gradually increased (0.54–1.05) and drought events mainly occurred in the southwest plateau and central mountainous regions (24–47 times), means that drought meteorological hotspots were mainly concentrated in the Sichuan basin. (3) The MLR indicated altitude (*H*) is not the main influencing factor that causes the spatial unevenness of precipitation in Sichuan Province, but altitude (*H*), temperature (*T*), longitude (*L*_o_) and latitude (*L*_a_) can co-determined the precipitation. The results of this study are instructive and practical for drought assessment, risk management and application decision-making in Sichuan Province, and have guiding significance for agricultural disaster prevention, mitigation and agricultural irrigation in Sichuan Province.

## Introduction

Drought has been one of the major natural disasters faced by human beings since ancient times. With the continuous warming of global climate, the frequency and intensity of drought events have gradually increased, which seriously affects the survival of human beings and the sustainable development of society, from 1950 to 2000, the average disaster area in China was about 21.14 million hectares, accounting for 14.9 percent of the country's sown area. 16.09 million people had difficulty in drinking water, and agricultural economic losses reached 6.64 billion yuan^1,2^. Wilhite and Glantz (1985) pointed out that drought usually occurs when an area does not get enough precipitation for a long time, leading to water shortages^3^. In a region where severe drought occurs continuously, the influencing factors and physical mechanisms are extremely complex, mainly due to the following factors: one is the backward water conservancy project facilities and insufficient disaster resistance; the other is the ecological over-cutting of forest resources which leads to serious soil erosion and vegetation destruction, which changes the natural regulation of the water cycle system, especially the scale, time, and frequency of the water cycle; third, abnormal atmospheric circulation leads to anomaly precipitation^4^. Previous research have shown that drought is mainly caused by insufficient rainfall and sharp rise in temperature^5^. Therefore, drought characteristics in a certain region and time scale can be evaluated according to the rainfall and temperature, and corresponding measures can be taken to reduce the frequency and intensity of drought^6^.

The drought characteristic index needs to meet the requirement of large-scale long-term continuous quantitative measurement and accurate historical record calculation, the index was defined by the World Meteorological Organization (WMO) in 1992 as an index relating to long-term cumulative effects and abnormal water shortages^7^. Palmer drought severity index (PDSI) defined climatologically appropriate precipitation as water demand, and judged water loss status based on actual precipitation difference, which can not only consider the current water supply and demand situation, but also include the previous dry and wet conditions and the duration to the current drought conditions^8^. However, PDSI is simplicity in space and cannot accurately describe large-scale drought characteristic changes^9^. Mckee^10^ replaces PDSI with standardized precipitation index (SPI), which has well applicability in time, space and rainfall probability distribution, and can detect the characteristics of drought activity over a wide area on different time scales, the SPI determined that precipitation is the main factor affecting drought intensity and duration^11^. The standardized precipitation evapotranspiration index (SPEI) is similar to SPI, SPEI considers surface evapotranspiration due to the background of global warming, and replaces precipitation in SPI with the difference between monthly precipitation and potential evapotranspiration^12^. SPEI and SPI are commonly used in current research and can jointly evaluate drought characteristics in time and space in drought-prone areas.

Southwest China is often regarded as a typical drought research area because it is one of the most important grain producing areas in China, but drought occurs at least once a year, and severe drought occurs once every 5–10 years^13^. Some recent studies on Southwest China have shown that 65% of the regional droughts in parts of Southwest China have increased significantly in drought intensity and duration, especially in Sichuan Province, and extreme drought indicate that the trend coefficient from east to west in the southwest region is decreasing^14,15^. The reason is that the transfer of drought area is affected by latitude, land–ocean interaction and topography^16^. The geographical conditions of southwestern China are complex, and the spatial distribution of precipitation is extremely uneven^17^. The average annual rainfall in Sichuan Province is only 604.2 mm^18^. The main reason is that Sichuan Province is one of the most complex topographic regions in the world. Its southwest and northwest are composed of the Qinghai-Tibet Plateau and cross section mountains, which cause a basin is formed in the east area, the external airflow is not easy to enter, various reasons leading to the foehn effect^19^. At the same time, the population and social development of Sichuan Province exceeds the carrying capacity of local water resources, leading to frequent droughts in the area. Chao indicated that change of climate increase Sichuan Province of average temperature and diurnal temperature and caused significant change of yields in around 30% of the maize planting area, corn production has shown a significant decline in the past 30 years^20^. According to the China Meteorological Disaster Yearbook (CMA), Sichuan Province covering 370,000 hectares of farmland suffered severe drought, and the result shows that 4.8 × 10^6^ tons of grains were lost and 6.8 × 10^5^ hectares of crops were completely destroyed from mid-July to early September 2006^21^. Sichuan Province is a major drought-prone area in southwest China, analyzing the temporal and spatial distribution of drought in Sichuan Province can effectively prevent and control the frequency of drought events. According to the development characteristics of different cities, monitoring the spatial and temporal distribution of drought can formulate the joint disaster prevention countermeasures of various departments, such as strengthening the construction of farmland water conservancy infrastructure regionally, popularizing farmland water-saving technology locality, and adjusting agricultural planting structure periodically^8^.

Based on the geographical features of Sichuan Province, it is mainly composed of plateau, mountain, hill and plain, divide Sichuan Province into four regions based on elevation, slope change rate, cumulative ground curve height, elevation coefficient of variation and relative elevation difference. Slope change rate is the rate of slope variation in differential space, the cumulative ground curve height is the comprehensive expression of vertical curvature and horizontal curvature to describe the degree of surface distortion change, the elevation coefficient of variation reflects the amount of elevation change of each vertex of the grid in the analysis area, and the relative elevation difference represents the elevation difference between any two points on the ground in topographic feature analysis. The main research purpose in the article as follows: (1) Calculate SPI and SPEI at different time scales based on precipitation and evapotranspiration, and use Mann–Kendall test (M–K trend test) to determine the drought trend of different geographical features, (2) calculate drought intensity, drought frequency and precipitation trend coefficient to analyze the spatiotemporal distributions of drought hotspots in Sichuan Province, (3) analyze the influencing factors leading to the difference of drought precipitation trend among different topography by using multivariable linear regression method (MLR). The results may be a reliable reference for the government to improve the specificity of drought prevention areas when considering ecological protection in the future.

## Materials and methods

### Study area

**Figure 1:**
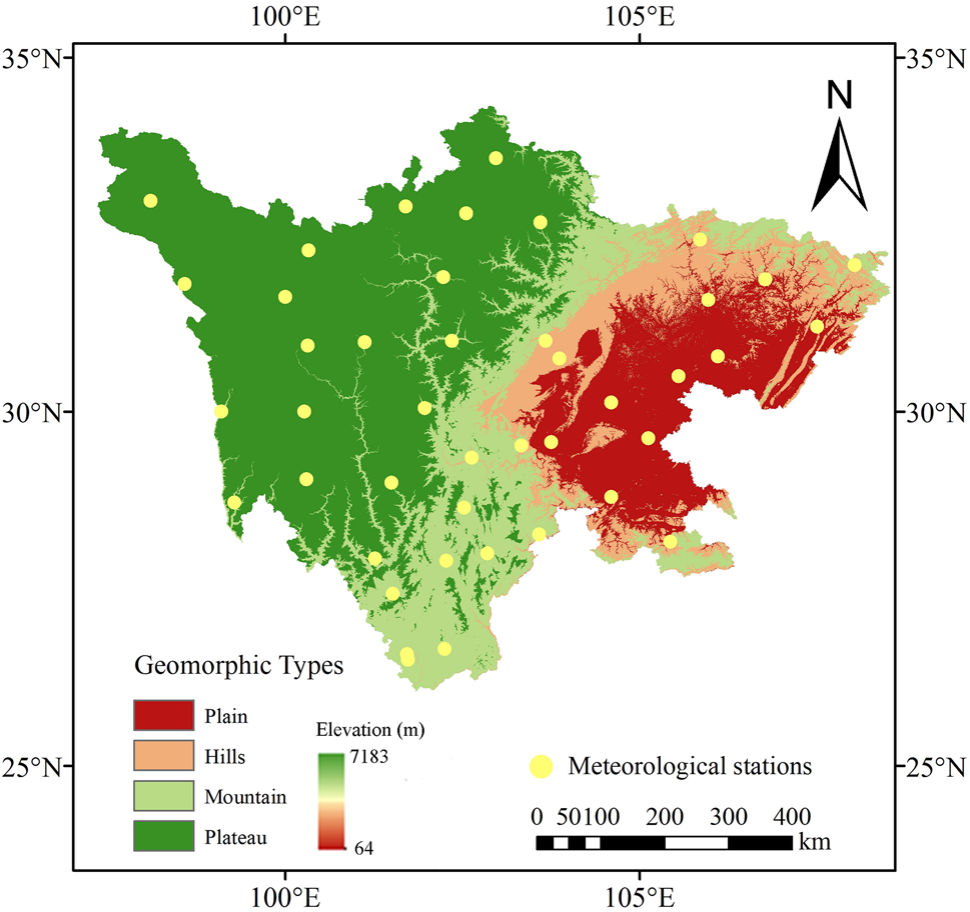
The location of the Sichuan Province and the distribution of meteorological stations. (The figure was generated by ArcGIS 10.6 software, https://desktop.arcgis.com/en/).

Sichuan Province is located in southwest China, on the upper reaches of the Yangtze River, between 92°21′ ~ 108°12′ east longitude and 26°03′ ~ 34°19′ north latitude, covering a total area of 4.8 × 10^5^ km^2^ and jurisdiction over 21 cities (states) and 183 counties (cities, districts), the Province’s registered population was 90.9 million in 2019^22^. The physiognomy varies differently from east to west, and the topography is complex and diverse. Sichuan is located at the first and second levels of the three major ladders of China's mainland topography, that is, in the transition zone between the first-level Qinghai-Tibet Plateau (3000–5000 m) and the second-level plain of the middle (1000–2000 m) and lower reaches of the Yangtze River, there are obvious characteristics of high west and low east^23^. The physiognomy type map of Sichuan Province is formed after classification and recoding function and five best factors (elevation, slope change rate, cumulative ground curve height, elevation coefficient of variation and relative elevation difference) clustering (Fig. 1). Eastern Sichuan is mainly plateaus and mountains, which altitude is above 3000 m and covers 3.2 × 10^5^ km^2^ approximately (67.3%). The eastern Sichuan basin is one of the four major basins in China, covering an area of 1.65 × 10^5^ km^2^ and located between alternating mountain areas. The eastern of Sichuan is hilly and plain and covering an area of 1.5 × 10^5^ km^2^ (32.7%), the elevation of the terrain decreases from west to east. There are large regional differences in rainfall distribution in Sichuan Province. The annual rainfall can reach more than 1700 mm at most and less than 400 mm at least. The annual rainfall in the middle and lower reaches of the Yangtze River is relatively uniform, reaching 1000 ~ 1800 mm^24^.

### Climate data

The data in this study are from China Meteorological Science Data Sharing Service System (http://cdc.cma.gov.cn), and the monthly precipitation data of 44 meteorological stations with long data series from 1961 to 2019 are selected, and which include precipitation (mm), air temperature (℃), wind speed (m s^−1^), relative humidity (%), and sunshine duration (h). The 44 data series is well represented, with few missing records and detection period longer than 50 year (44 series have full data during the period 1961 ~ 2019). There were 3 data absence sites, accounting for 6.82% of the total data, and the absence time was part of the months between 1969 and 1970. Based on the locations of the meteorological stations, we interpolated the data at a resolution of 1 km by means of inverse distance weighted (IDW) interpolation to obtain the spatial distribution of drought and passed the homogeneity test. The research scope and the distribution of each site are shown in Fig. 1.

### Standardized precipitation index (SPI)

Standardized precipitation index (SPI) is an indicator representing the probability of rainfall occurrence in a certain period of time in a region. It has the advantages of simple calculation and stability, and eliminates the temporal and spatial difference of rainfall. It is sensitive to drought change and applicable to drought monitoring and assessment of climatic conditions above the monthly scale. Mckee^10^ proposed the SPI in 1993 and used it to assess climate and drought change, an incomplete gamma probability density function is firstly fitted to a given frequency distribution of precipitation series:

1 $$ g(x) = \frac{1}{{\beta^{\alpha } \Gamma (a)}}x^{\alpha - 1} e^{{ - \frac{x}{\beta }}} ,x{ > }0 $$

where α is a parameter about shape, β is a parameter about scale, x is the amount of precipitation, and the gamma function is presented as:

2 $$ \Gamma (\alpha ) = \int_{0}^{\infty } {x^{\alpha - 1} e^{ - x} dx} $$

the best values of α and β are estimated by the maximum likelihood method.

3 $$ \hat{\alpha } = \frac{1}{4A}\left( {1 + \sqrt {1 + \frac{4A}{3}} } \right) $$

4 $$ \hat{\beta } = \frac{{\overline{x}}}{{\hat{\alpha }}} $$

5 $$ A = \ln (\overline{x}) - \frac{{\sum {\ln (x)} }}{n} $$

where n is the number of precipitation series.

The cumulative probability for a given month then can be obtained by the following equation:

6 $$ G(x) = \int_{0}^{x} {g(x)} dx = \frac{1}{{\beta^{\alpha } \Gamma (\alpha )}}\int_{0}^{x} {x^{\alpha - 1} e^{ - x/\beta } dx} $$

the SPI can be calculated as follows:

7 $$ SPI = S\frac{t - (c2t + c10) + c0}{{\left[ {\left( {d3t + d2} \right)t + d1} \right]t + 1.0}} $$

8 $$ t = \sqrt {\ln \frac{1}{{G\left( x \right)^{2} }}} $$

where x is the amount of precipitation and G(x) is Γ function related precipitation probability distribution, S is the positive and negative coefficient of cumulative probability distribution, when G(x) > 0.5, S = 1 and when G(x) ≤ 0.5, S = -1. c_0_ = 2.5155, c_1_ = 0.8028, c_2_ = 0.0103, d_1_ = 1.4327, d_2_ = 0.1892, d_3_ = 0.0013.

### Standardized precipitation evapotranspiration index (SPEI)

Standardized precipitation evapotranspiration index (SPEI) replaces the monthly rainfall in SPI with the difference between monthly rainfall and monthly potential evapotranspiration, and takes into account the temperature factor, and introduces the influence of surface evaporation changes, which is more sensitive to the drought reaction caused by global temperature rise.

In order to estimate the value of SPEI, the difference of the water balance is normalized as log-logistic probability distribution. The following equation expresses the probability density function:

9 $$ f(x) = \frac{\beta }{\alpha }\left( {\frac{x - \gamma }{\alpha }} \right)\left[ {1 + \left( {\frac{x - \gamma }{\alpha }} \right)} \right]^{ - 2} $$

where parameters α, β, and γ represent scale, shape and origin, respectively. Therefore, the probability distribution function can be expressed as:

10 $$ F(x) = \left[ {1 + \left( {\frac{\alpha }{x - \gamma }} \right)^{\beta } } \right]^{ - 1} $$

Vicente–Serrano^25^ calculated the SPEI as follow:

11 $$ SPEI = W - \frac{{C0 + C1W + C2W^{2} }}{{1 + d1W + d2W^{2} + d3W^{3} }} $$

When *P* ≤ 0.5, $W = \sqrt { - 2\ln (P)}$, and when P > 0.5, $W = \sqrt { - 2\ln (1 - P)}$, C_0_ = 2.5155,C_1_ = 0.8028, C_2_ = 0.0203, d_1_ = 1.4327, d_2_ = 0.1892, d_3_ = 0.0013. The categorization of drought classified by the SPI and SPEI is show in Table 1.

**Table 1:** Climatic moisture categories for the SPI and SPEI^2^.

Climatic moisture categories	SPI or SPEI
Extremely wet	≥ 2.0
Severely wet	1.5 to 1.99
Moderately wet	1.0 to 1.49
Normal	0.99 to − 0.99
Moderate drought	− 1.0 to − 1.49
Severe drought	− 1.5 to − 1.99
Extreme drought	≤ − 2.0

### Runs theory and conditional probability

Drought characteristics include drought duration, drought intensity and drought frequency. When calculating the absolute value of SPI/SPEI, the value of SPI/SPEI under normal conditions (SPI/SPEI ≥ − 1) is also calculated in general method, which will greatly affect the assessment of drought. So we used the runs theory proposed by Yevjevich^26^ to define the drought intensity and drought frequency, the runs theory defines a part of the drought variable time series in which all values are lower or higher than the selected threshold, called negative or positive run, the drought intensity calculation formula is as follows:

12 $$ S = \frac{{\sum\limits_{n = 1}^{T} {\left| {\left. {SSPI/SPEI - K} \right|} \right.} }}{T} $$

where *S* is drought intensity, *S*_SPI/SPEI_ is SPI or SPEI value below the threshold, *K* is drought threshold, set to be less than or equal to -1 in this study, means the drought level is greater than moderate drought, T is the duration of the drought process. Drought frequency is used to assess the frequency of drought in the area, the formula is as follows:

13 $$ P = \frac{n}{N} \times 100\% $$

where N represents the time period of site detection, n represents the number of droughts at the site during the time period.

Conditional probability (C*p*) refers to the probability of occurrence of a given event A under the condition that another event B has occurred, which means C*p* (A/B). In this study, C*p* refers probability of the event probability of SPI_drought_ in another event probability of SEPI_drought_, which record C*p* (SPI), on the contrary record C*p* (SPEI), the formula is as follows:

14 $$ Cp{\text{(SPI)}} = \frac{TSPI \, /SPEI}{{TSPEI}}\;\;\;{\text{or}}\;\;\;Cp(SPEI) = \frac{TSPEI/SPI}{{TSPI}} $$

where T_SPI_ and T_SPEI_ represents the the times of droughts in an area in a period of time based on the SPI/SPEI value, T_SPI/SPEI_ and T_SPEI/SPI_ represents based on the SPEI/SPI assessment that drought has occurred, SPI/SPEI re-evaluate the times of droughts in the area.

### M–K trend test and multivariable linear regression method

Theil–Sen Median method was used to calculate the trend value, which was usually combined with MK non-parametric test, the MK method was used to determine the significance of the trend. Theil–Sen Median method was a robust non-parametric statistical trend calculation method, which had high computational efficiency and was not sensitive to measurement error and outlier data, and was often used in trend analysis of long time series data, the formula is as follows:

15 $$ \beta = mean\left( {\frac{xj - xi}{{j - i}}} \right),\forall j{ > }i $$

where, x_j_ and x_i_ are time series data, β greater than 0 means the time series presents an upward trend, while less than 0 means the time series presents a downward trend.

The M–K trend test is a non-parametric statistical test method. Its advantage is that the measured values do not need to follow normal distribution and are not affected by missing values and outliers. It is widely used in the trend significance test of long time series data, and its statistical method is as follows^27^:

16 $$ z = \left\{ {\begin{array}{*{20}l} {\frac{S}{{\sqrt {Var(S)} }}} \hfill & {(S > 0)} \hfill \\ 0 \hfill & {(S = 0)} \hfill \\ {\frac{S + 1}{{\sqrt {Var(S)} }}} \hfill & {(S < 0)} \hfill \\ \end{array} } \right. $$

17 $$ S = \sum\limits_{i = 1}^{n - 1} {\sum\limits_{j = i + 1}^{n} {sign(xj - xi)} } $$

18 $$ sign(\theta ) = \left\{ {\begin{array}{*{20}l} 1 \hfill & {(\theta > 0)} \hfill \\ 0 \hfill & {(\theta = 0)} \hfill \\ { - 1} \hfill & {(\theta < 0)} \hfill \\ \end{array} } \right. $$

$$ E(S) = 0 $$

19 $$ Var(S) = \frac{n(n - 1)(2n + 5)}{{18}} $$

where, x_j_ and x_i_ are time series data, When n ≥ 8, the test statistic S is approximately normally distributed, and its mean value and variance are as follows: at a given significance level α, if |z|> z_1-α/2_, means assumption that there is no trend is rejected, The time series data have obvious trend change, z_1−α/2_ is the value corresponding to the standard normal function distribution table at the confidence level α. When |z| is greater than 1.65, 1.96 and 2.58, it indicates that the trend has passed the significance test with a reliability of 90%, 95% and 99% respectively. In this study, the M–K trend test was used to determine the drought characteristic trend of different physiognomy on different time scales (1 month, 3 month, 6 month and 12 month) and the drought trend changes of precipitation at different time periods of each station.

The multivariable linear regression method (MLR) is to apply mathematical statistics to establish a multiple regression model in which meteorological elements influence the spatial interpolation factors of meteorological elements. We set precipitation (*Y*) as the dependent variable, and the four independent variables that affect precipitation are altitude (*H*), temperature (*T*), longitude (*L*_o_) and latitude (*L*_a_) respectively. We assume that the influence of each independent variable on the dependent variable is linear, the mean value of precipitation varies uniformly with the change of the independent variable when the other independent variables remain unchanged. In this study, a multiple regression model of precipitation (*Y*) for altitude (*H*), temperature (*T*), longitude (*L*_o_) and latitude (*L*_a_) was established, and the residuals were calculated, the expression is as follows:

20 $$ Y\left( {H,T,Lo,La} \right) = b0 + b1H + b2T + b3Lo + b4La + \varepsilon $$

where *b*_*1*_, *b*_*2*_, *b*_*3*_, *b*_*4*_ is the undetermined coefficient, ɛ is the residual value and b_0_ is the constant^28^.

## Result

### Multi-scale patterns of the drought

**Figure 2:**
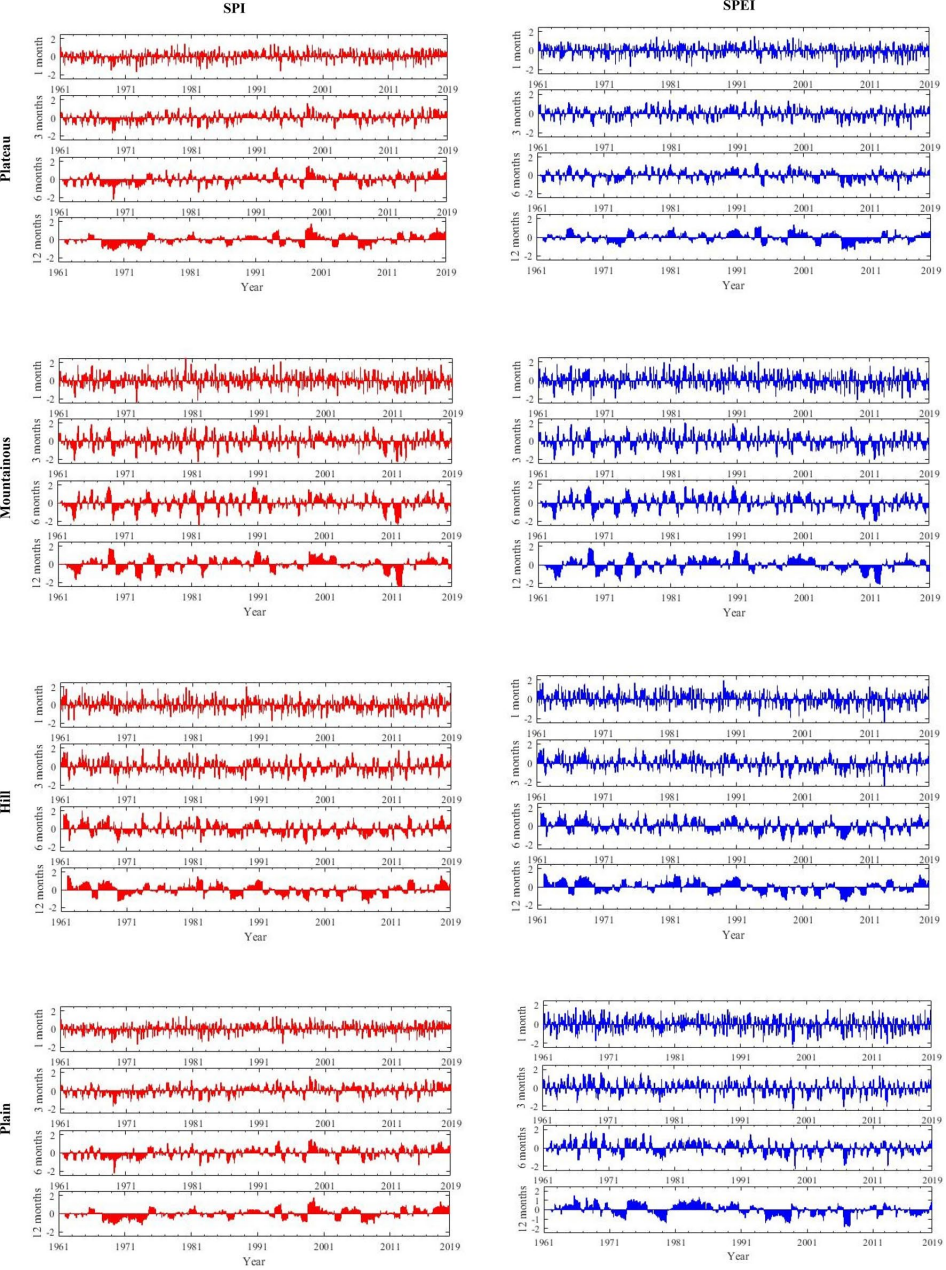
The SPI and SPEI at different timescales for different geomorphologic types (plateaus, mountains, hills and plains) in Sichuan Province. (The figure was generated by MATLAB 2016 software, https://www.mathworks.com/products/matlab.html).

The monthly SPI and SPEI was calculated at 4 time scales (1 month, 3 months, 6 months and 12 months) for different physiognomy (Plain elevation range was 0–200 m, hilly elevation range was 200–500 m, fluctuation was not more than 200 m, mountain elevation was 500–1000 m, fluctuation is not more than 200 m, plateau 1000 m above, fluctuation was more than 200 m) during the period 1961 to 2019 (Fig. 2). Then, these SPI and SPEI value at 4 time scales were averaged to characterize the drought conditions in Sichuan Province, China. There are significant differences in the sensitivity of SPI and SPEI values at different time scales, the smaller the time scale, the more obvious the wet and dry changes. The SPEI value of the Sichuan plateau, mountain, hill and plain all shows an downward trend on various time scales, but SPI value of Sichuan Plateau and mountain shows a upward trend on various time scales, which are calculated by M–K test (Table. 2). Because SPEI takes into account evapotranspiration, it is more sensitive to changes in drought, so there are some differences with SPI. This study used conditional probability (C*p*) to analyze the difference between SPI and SPEI, we defined the degree of moderate drought above (SPI/SPEI ≤ − 1) as an event of drought, C*p* refers probability of the event probability of SPI_drought_ in another event probability of SPEI_drought_, which record C*p* (SPI), on the contrary record C*p* (SPEI), The C*p* (SPI) and C*p* (SPEI) of different landforms at each time scale are shown in Table 3. In the hilly areas, C*p* (SPI) was 0.55, 0.53, 0.62, 0.58 and C*p* (SPEI) was 0.67, 0.68, 0.71, 0.64 in 1-month time scale from 1961 to 2000, and the difference in the results was not significant (*P* > 0.05), it means that SPI and SPEI can mutually confirm drought conditions in a certain time and area. However, there are significant differences in drought characteristics between SPI and SPEI in some regions and time periods (*P* < 0.05), for example, in the plateau region from 1961 to 2019, the C*p* (SPI) decreases continuously while the C*p* (SPEI) increases continuously (C*p* (SPI) was 0.58, 0.33, 0.25, 0.25, 0.28, 0.33 and C*p* (SPEI) was 0.66, 0.53, 0.84, 0.33, 0.55, 0.98 in 3-month time scale from 1961 to 2019), and C*p* (SPEI) was greater than C*p* (SPI) on the time scale of all regions. The results showed that SPEI was more sensitive to drought assessment than SPI, and could more accurately reflect dry/wet alternations in more complex regions.

**Table 2:** MK Trend test coefficient and significance coefficient (*P*) of SPI and SPEI in different geomorphologic types in Sichuan province on different time scales.

Physiognomy	SPEI	Trend (year^−1^)	Z	*P*	SPI	Trend (year^−1^)	Z	*P*
Plateau	SPEI-1	− 0.0140	− 1.3695	0.171	SPEI-1	0.0444	− 4.7993	0.000
	SPEI-3	− 0.0182	− 1.7473	0.081	SPEI-3	0.0713	− 7.4826	0.000
	SPEI-6	− 0.0084	− 0.8547	0.393	SPEI-6	0.0849	− 8.9138	0.000
	SPEI-12	− 0.0106	− 1.1072	0.268	SPEI-12	0.0875	− 9.3591	0.000
	SPEI-1	− 0.0264	− 1.8070	0.071	SPEI-1	0.0035	− 0.2733	0.785
Mountainous	SPEI-3	− 0.0310	− 2.1756	0.030	SPEI-3	0.0098	− 0.7259	0.468
	SPEI-6	− 0.0204	− 1.4790	0.139	SPEI-6	0.0201	− 1.5957	0.111
	SPEI-12	− 0.0091	− 0.7693	0.442	SPEI-12	0.0324	− 2.6695	0.008
	SPEI-1	− 0.0389	− 3.0682	0.002	SPEI-1	− 0.0111	− 0.8880	0.375
Hill	SPEI-3	− 0.0601	− 4.6623	0.000	SPEI-3	− 0.0217	− 1.6395	0.101
	SPEI-6	− 0.0699	− 5.5934	0.000	SPEI-6	− 0.0277	− 2.2546	0.024
	SPEI-12	− 0.0795	− 6.4637	0.000	SPEI-12	− 0.0412	− 3.2525	0.001
	SPEI-1	− 0.0348	− 2.5763	0.010	SPEI-1	− 0.0154	− 1.2187	0.223
Plain	SPEI-3	− 0.0530	− 3.9837	0.000	SPEI-3	− 0.0278	− 2.2332	0.026
	SPEI-6	− 0.0644	− 5.4286	0.000	SPEI-6	− 0.0364	− 3.2093	0.001
	SPEI-12	− 0.0936	− 8.5257	0.000	SPEI-12	− 0.0557	− 5.4968	0.000

**Table 3:** Conditional probability of SPI and SPEI (C*p* (SPI) and C*p* (SPEI)) in different geomorphic types in Sichuan province in different periods and time scales.

Physiognomy	Period	C*p* (SPI)				C*p* (SPEI)			
		1	3	6	12	1	3	6	12
Plateau	1961–1970	0.33	0.58	0.75	/	0.29	0.66	0.94	/
	1971–1980	0.41	0.33	0.49	0.43	0.45	0.53	0.51	0.56
	1981–1990	0.82	0.25	0.66	/	0.83	0.84	0.33	/
	1991–2000	0.95	0.25	0.33	/	0.54	0.33	0.25	/
	2001–2010	0.14	0.28	0.44	0.11	0.95	0.55	0.66	0.87
	2011–2019	0.44	0.33	0.5	/	0.95	0.98	0.96	/
Mountainous	1961–1970	0.64	0.82	0.75	0.78	0.98	0.93	0.92	0.95
	1971–1980	0.51	0.81	0.82	0.95	0.33	0.75	0.97	0.93
	1981–1990	0.56	0.86	0.72	0.22	0.71	0.66	0.73	0.25
	1991–2000	0.71	0.75	0.57	/	0.71	0.97	0.55	/
	2001–2010	0.29	0.63	0.63	0.92	0.83	0.98	0.87	0.98
	2011–2019	0.59	0.64	0.76	0.97	0.91	0.9	0.91	0.99
Hill	1961–1970	0.55	0.8	0.92	/	0.67	0.81	0.95	/
	1971–1980	0.53	0.61	0.93	/	0.68	0.66	0.56	/
	1981–1990	0.62	0.54	/	/	0.71	0.59	/	/
	1991–2000	0.58	0.69	0.63	0.92	0.64	0.95	0.78	0.96
	2001–2010	0.57	0.7	0.64	0.92	0.89	0.95	0.96	0.96
	2011–2019	0.64	0.62	0.43	/	0.98	0.83	0.67	/
Plain	1961–1970	0.44	0.85	0.91	/	0.57	0.95	0.83	/
	1971–1980	0.83	0.75	0.57	0.33	0.95	0.75	0.67	0.95
	1981–1990	0.44	0.75	/	0.89	0.95	0.95	/	0.96
	1991–2000	0.93	0.74	0.86	/	0.87	0.78	0.87	/
	2001–2010	0.67	0.69	0.76	0.92	0.91	0.95	0.91	0.95
	2011–2019	0.67	0.43	0.55	/	0.8	0.75	0.95	/

(‘/’ means that there is no drought, so the conditional probability cannot be calculated).

To study the overall trends of SPEI and SPI in Sichuan Province, the non-parametric M–K test method was used to analyze the drought trends of different landform types on different time scales from 1961 to 2019 (Table 2). Base on the trend results, we can see that the drought conditions of hilly and plain areas continued to aggravate from 1961 to 2019, and their SPEI showed an extreme significant downward trend on all time scales (*P* < 0.01), and partly SPI trends indicated a significant downward trend (*P* < 0.05), the drought trend was gradually increasing when SPI/SPEI calculated with more lagged time scales. The SPEI drought trend in plain and hilly regions was greater than that in plateau and mountain regions on all time scales (− 0.039 year^−1^ for 1-month in hilly, − 0.035 year^−1^ for 1-month in plain,  − 0.14 year^−1^ for 1-month in plateau, − 0.026 year^−1^ for 1-month in mountain), which means that the degree of drought in the eastern part of Sichuan Province is increasing, and the drought trend is gradually shifting from the southwest to the east.

### Drought precipitation trend

**Figure 3:**
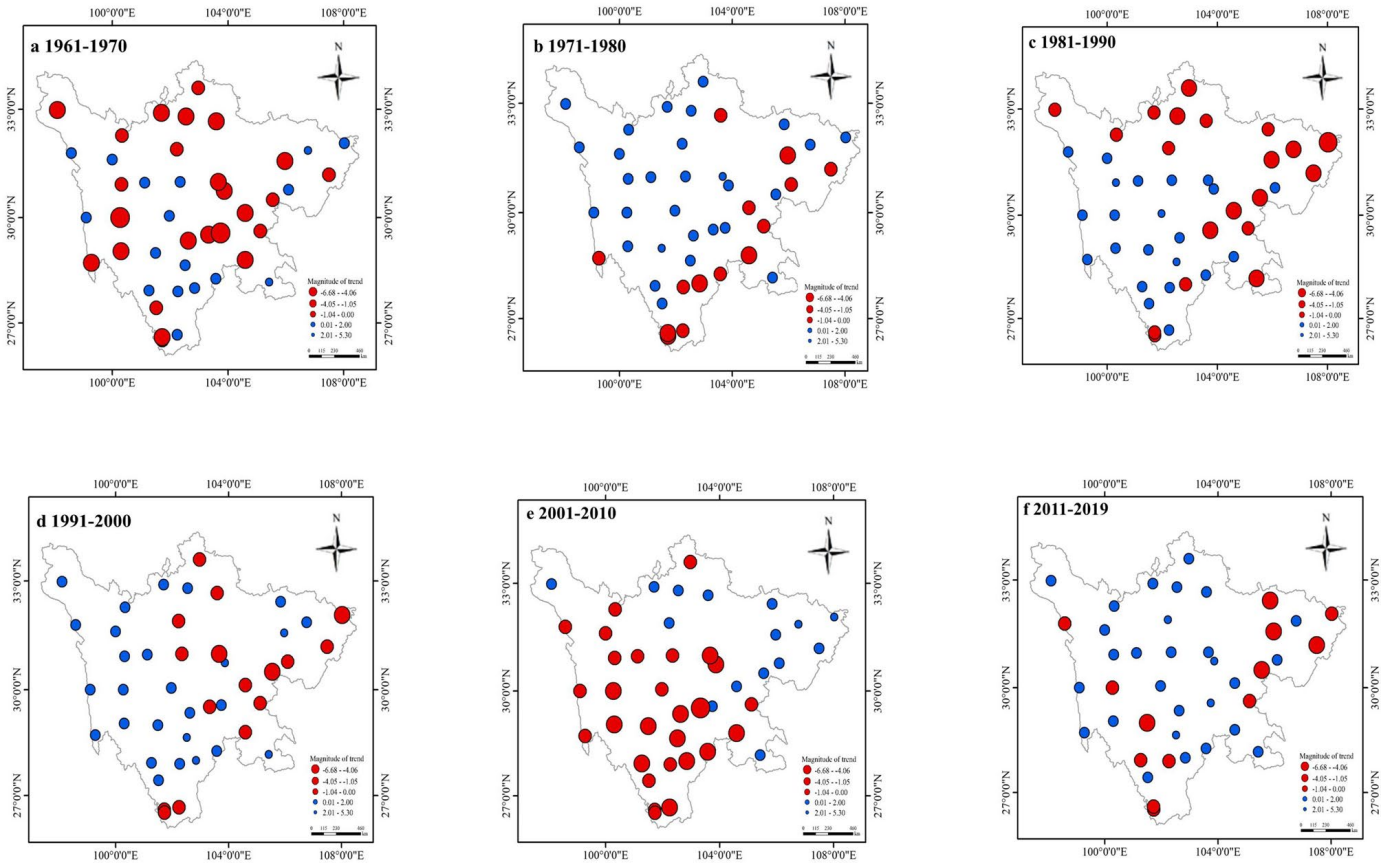
MK Trend test coefficient of precipitation in Sichuan province on different time scales (1961–1970s, 1971–1980s, 1981–1990s, 1991–2000s, 2001–2010s, 2011–2019s), red represents a negative trend and blue represents a positive trend. (The figure was generated by ArcGIS 10.6 software, https://desktop.arcgis.com/en/).

To further analyze the trend of drought characteristics in Sichuan Province from 1961 to 2019, as shown in Fig. 3, we analyzed the meteorological changes of 44 stations in Sichuan Province in different periods (1961–1970s, 1971–1980s, 1981–1990s, 1991–2000s, 2001–2019s) through the non-parametric MK test method based on precipitation data. In the period 1961–1970s, 47.7% of stations presented decreasing trends and only 16.7% of the sites showed significance (*P* < 0.05), 55.5% plateau, 75.5% hilly and 65.5% plain sites all show negative trends and the highest magnitudes trend in plateau areas was − 4.37 year^−1^. In the period 1971–1980s, the drought trend in the all area gradually alleviated, and only 29.5% of the sites showed a negative trend, which was not significant (*P* > 0.05), the magnitude of trend varied between − 2.2 to 2.0 year^−1^. However, during 1981–1990s, the rainfall in the eastern part of Sichuan gradually decreased, average monthly rainfall at the site is only 95.5 mm, and the drought trend showed a significant negative trend, and 80% of the stations in the eastern region showed a negative growth, the magnitude of trend varied between − 4.4 to 0.1 year^−1^ in eastern region, partly plateau areas (11%) have also begun to show negative trends. During the period from 1991 to 2000s, the drought trend in the eastern plain of Sichuan gradually slowed, and 34% of the stations showed a negative growth trend but none was significant (*P* > 0.05), and the magnitude of trend varied between − 3.3 to 2.8 year^−1^. While in the period 2001–2010s, the mountainous and hilly regions in central Sichuan showed a negative trend, with an amplitude ranging from − 0.37 to − 4.17 year^−1^, and the rainfall in the mountainous and hilly regions decreased significantly (*P* < 0.05), and the monthly average rainfall was only 71.2 mm. From 2011 to 2019s, most sites in Sichuan Province showed positive trends, only 27.3% of sites showed a slight negative trend, partly sites in mountainous regions have the largest magnitude values (5.3 year^−1^) from 1961 to 2019.

The percentages of stations with negative rainfall trends in each period (1961–1970s, 1971–1980s, 1981–1990s, 1991–2000s, 2001–2009s, 2010–2019s) were 43.6%, 29.5%, 43.2%, 34.1%, 59.1% and 27.3%, the reason for the increase in the ratio of drought stations in 1961–1970s, 1981–1990s and 2000–2009s was due to the extreme drought events in southwest China in 1961s and 2006s. In the early years, both the northwest and eastern regions showed a negative growth trend of rainfall. However, since the 1980s, the drought trend in the eastern regions of Sichuan Province has gradually intensified, while the trend in the northwest has gradually slowed and gathered in the mountainous regions of central Sichuan in the early 2000s. The drought precipitation trend demonstrated decreasing trends transfer from northwest to east and gather in central Sichuan Province.

### Drought intensity

**Figure 4:**
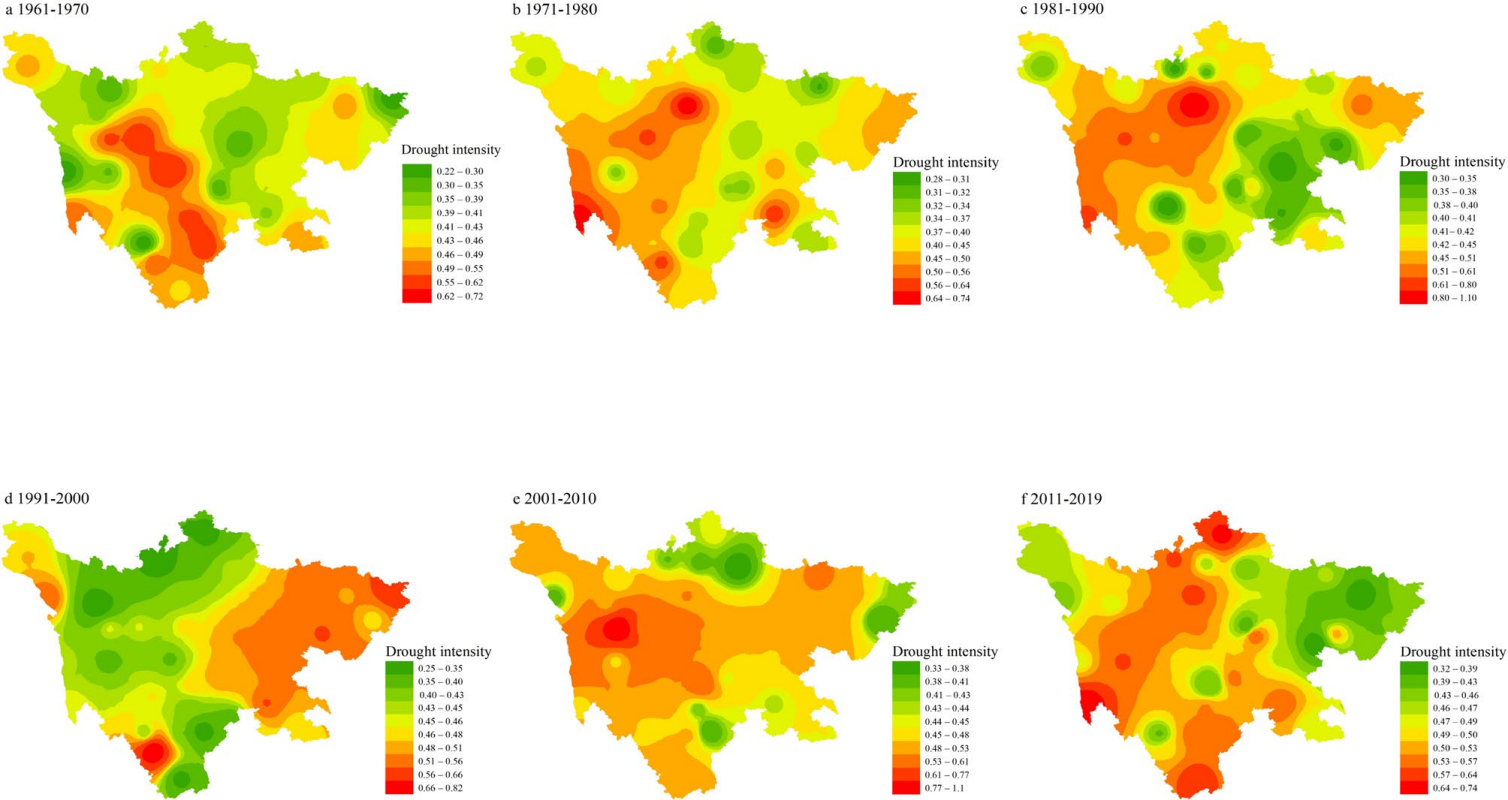
Base on 1-month SPEI, drought intensity during individual decades (1961–1970s, 1971–1980s, 1981–1990s, 1991–2000s, 2001–2010s, 2011–2019s) and for the full period 1960–2016 in Sichuan Province, China. (The figure was generated by ArcGIS 10.6 software, https://desktop.arcgis.com/en/).

Based on SPEI was more sensitive to drought assessment than SPI on characterize the temporal and spatial differences of droughts, meanwhile Li^29^ proved the 1-month times scale for SPEI was adopted as a good indicator of changes in drought, the spatial distribution diagram of the inter-annual variation of drought intensity was calculated by the run theory and 1-month SPEI (Fig. 4). In the 1960s, drought areas were mainly distributed in the southwest of Sichuan plateau region and the central Sichuan basin region, the maximum drought intensity was 0.72 and 0.68, respectively. In the 1970s and 1980s, drought mainly occurred in the northwest plateau region, the drought intensity range was 0.52–0.7 and 0.49–0.64 respectively, but the degree of drought gradually relieved in the central Sichuan basin, and its intensity ranged from 0.28 to 0.33. In the eastern plains and hilly regions, the degree of drought increased significantly in the 1990s, with the range of drought intensity rising from 0.28–0.45 to 0.46–0.66. However, from 2000 to 2009, the drought region shifted from the eastern plain region to the western plateau region, and the drought intensity in the eastern part of Sichuan gradually weakened (0.36–0.51), while the drought intensity in the western region gradually increased (0.54–1.05). By 2019, the southwest and northwest fringe areas of Sichuan have become drought-prone areas, with the drought intensity being 0.74 and 0.62 respectively.

Sichuan Province is surrounded by the Qinghai-Tibet Plateau, Qinling Mountains and Yunnan-Guizhou Plateau, it is difficult for external air flow to enter and release internal hot air^30^. At the same time, the western plateau area has caused the southeast monsoon to sink in central Sichuan, causing frequent droughts in the Yangtze River coast. The southeast monsoon from east to west gradually sink into the Sichuan basin, its rainfall from the east to the west in turn decline, as result, there are frequent occurrences of drought in plateau and mountainous regions. The drought between 2000 and 2009 mainly occurred in eastern Sichuan because of the extreme drought in Sichuan Province in 2006^19^. The South Asian High and the subtropical regions of the Western Pacific were active, which caused the eastern Sichuan to be controlled by the continental high temperature. The eastern plain area of Sichuan province is a dense area of human activities, over-exploitation of resources, lack of water conservancy construction, and natural and human factors have caused drought to shift to the east. The trend of drought in the early twentieth century corresponded to rising levels of greenhouse gas emissions. Aerosols formed by the atmosphere and suspended solid and liquid particles affect rainfall and change cloud cover, so human economic activities are closely related to the risk of drought.

### Drought meteorological hotspots frequency

**Figure 5:**
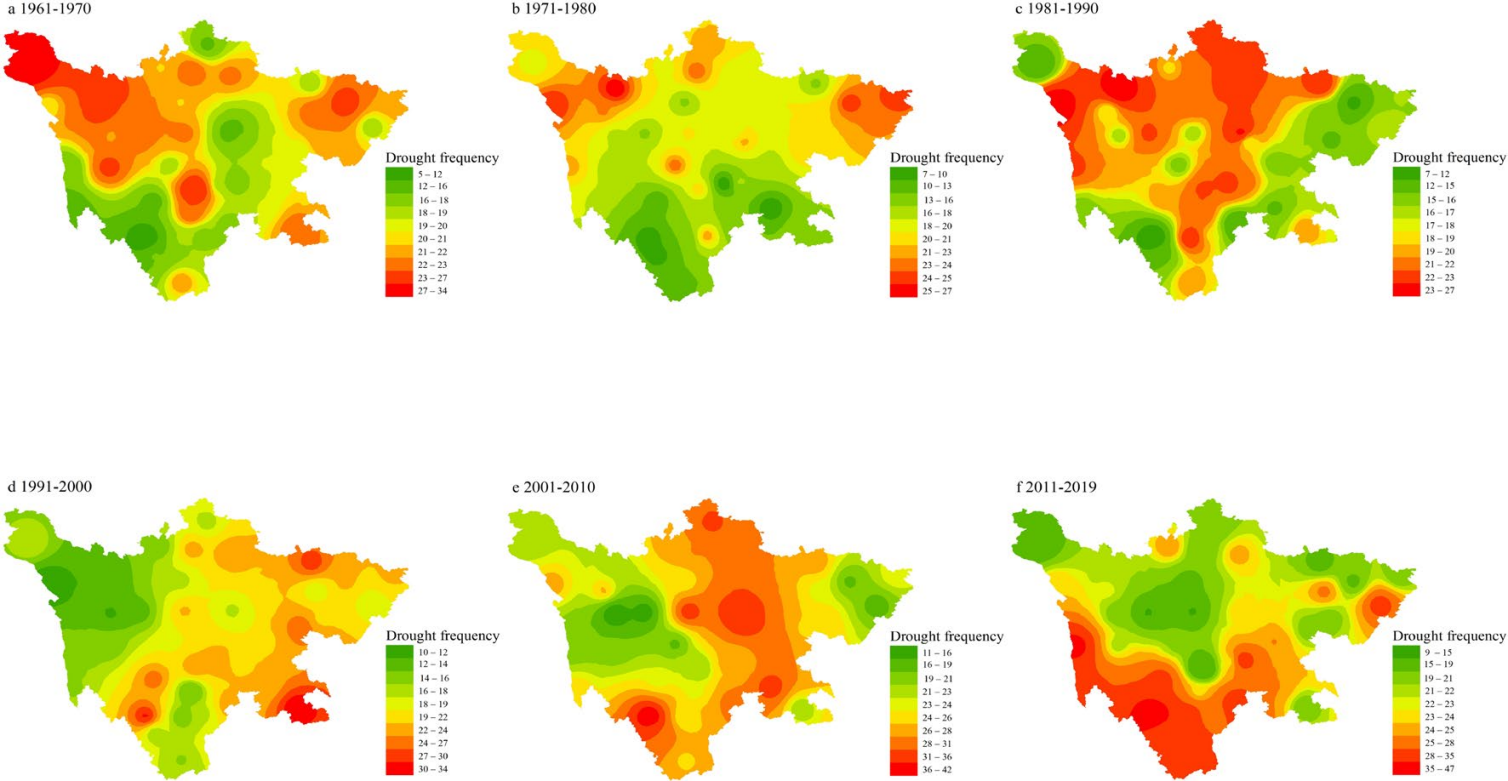
Base on 1-month SPEI, drought frequency during individual decades (1961–1970s, 1971–1980s, 1981–1990s, 1991–2000s, 2001–2010s, 2011–2019s) and for the full period 1960–2016 in Sichuan Province, China. (The figure was generated by ArcGIS 10.6 software, https://desktop.arcgis.com/en/).

Based on the monthly SPEI values of 44 meteorological stations in Sichuan Province from 1961 to 2019, and calculate the frequency of moderate drought and above drought at each station, we identify drought meteorological hotspots by drought frequency (Fig. 5). The results show that from 1961 to 2019 in the 44 meteorological stations in Sichuan Province, the frequency of droughts above moderate drought at each station was between 13.6 and 19.1%, which means that most parts of Sichuan Province can occur once drought event every 5.2–7.3 years. In the period 1961–1970, drought frequency showed high values in Shiqu and Hanyuan county, where the frequencies were 34 and 31 time, geographically located on plateau and mountain respectively. In the period 1971–1980, the frequency of droughts in most parts of Sichuan Province has decreased, even in plateaus and mountainous landforms only 7–26 times have occurred. However, during 1981–1990, the frequency of droughts in central Sichuan increased significantly, and they mainly occurred in northwestern plateau, especially in areas such as Dege, Seda, and Dujiangyan, the drought frequencies were 26, 27, and 23 times respectively. In the period 1991–2000, the drought frequency in the northwest plateau dropped to 10–24 times, while the drought frequency in the eastern basin rose to 34 times in Yongxu county. After 2000, the frequency of drought increased significantly compared with 1961–1999, which mainly occurred in the southwest plateau and central mountainous regions, the drought frequency ranged from 24 to 47 times.

According to the results of drought frequency in 1961–2019, we found that the Drought meteorological hotspots were mainly concentrated in plateau and mountainous areas. The drought frequency has been between 7 and 27 times before 2000, and the drought frequency has increased significantly after 2000, reaching the highest in Muli (47 times). Comparing the drought duration data in Southwest China, we found that there were extreme drought events in Southwest China in 2006 and 2010. Statistics show that the disaster area of crops in Sichuan Province reached 5.1 × 10^5^ km, the disaster-affected area was 2.5 × 10^5^ km, and the no-harvest area was 5.7 × 10^4^ km. The affected population was 8.28 million and the economic loss was 1.38 billion yuan^21^. In the early 1960s, due to backward water conservancy projects and over-deforestation, serious soil erosion was caused, and the ecosystem and water circulation system were disordered and rainfall decreased. The extreme drought event in southwest China in 2006 was due to the weakening of the westerly circulation effect due to the abnormal subtropical high in the western Pacific, which affected the significant decrease in the intensity and frequency of southward cold air activity.

For the analysis of drought frequency factors in Sichuan Province, synoptic climatology was a major factor. we found that the anomalies of the Western Pacific Subtropical High and Continental Subtropical High, the northerly of the Western Pacific Subtropical High and the easterly of the South Asian High have made the downdraft in Sichuan stronger and inhibited the transportation of water vapor in the Bay of Bengal, it was the main reason for the severe drought in Sichuan in 2006. Sea temperature also has a certain impact on rainfall in Sichuan Province. The tropical western Pacific and Indian Ocean are warming up, causing an abnormal anticyclonic circulation over the tropical western Pacific, causing anomalous southwestern airflow and strengthening along the southeast coast of China, and sinking in the east of the plateau by the northwest. Airflow control has made it difficult for the water vapor in the Bay of Bengal to reach Sichuan Province, causing long-term lack of rainfall in the area. At the same time, the reason of drought prone areas of Sichuan province in addition to the geographical factors, Sichuan province is located in the basin, surrounded by the Tibetan plateau, Qinling and Yunnan–Guizhou plateau, the air is not easy to enter, and the internal air also is not easy to release, forming climate effect of bamboo steamer, south of the Yunnan–Guizhou plateau of Sichuan province made the southeast monsoon in the basin subsidence, along the Yangtze river region drought less rain, which making the drought aggravated.

## Discussion

Some studies have analyzed the causes of frequent droughts in Sichuan Province, and concluded that three major factors are the special climate caused by topography, the uneven temporal and spatial distribution of rainfall, and the abnormal atmospheric circulation^8,12,16,22^. Li using the unary linear regression trend analysis method to analyze the drought in Sichuan Province, it is found that the drought trend in the northwest region has gradually eased in the past ten years, while the drought in the Sichuan Basin and the western plateau area has gradually increased, and the drought trend changes between different terrains are significantly different^16^. The study found that the monthly average temperature of Sichuan for many years is negatively correlated with the latitude. At the same time, the longitude of the average temperature distribution in Sichuan is much greater than the latitude zonality, and the monthly average temperature for many years is negatively correlated with the altitude, indicating that the temperature, latitude, longitude and altitude are main factors affecting drought and rainfall. In addition to the factors caused by the internal topography of Sichuan Province, the atmospheric circulation is also a direct cause of abnormal weather and climate. Antonio found that active convective activities in the subtropical region of the western Pacific can cause the western continental sinking airflow to prevail, rainfall is suppressed, and the temperature is abnormally high^31^.

Sichuan Province has significant regional differences in climate and is dominated by vertical climate. The eastern part of Sichuan Province belongs to the subtropical climate region, while the western high principle belongs to the Qinghai-Tibet Plateau climate region^32^. The subtropical climate is mainly concentrated in the mountainous river region and the bottom area of the basin, both of which are dominated by the vertical climate^33^. Above the mountain, there is a temperature or even a frigid climate, with large vertical differences in space. The climatic area of the Qinghai–Tibet Plateau is dominated by sub-frigid climate, and the altitude difference causes the vertical distribution to be more obvious than that of mountainous areas^34^. The complex spatial distribution of rainfall in Sichuan Province, which is not only restricted by atmospheric conditions but also affected by topography and altitude. We use multivariable linear regression method (MLR) to establish a multiple regression model of average annual precipitation in different periods based on altitude (*H*), temperature (*T*), longitude (*L*_*o*_) and latitude (*L*_*a*_), and calculate its significance coefficients (Table 4). The results show that altitude, temperature, longitude and latitude can significantly affect the average annual precipitation in each period. Precipitation and altitude, temperature and longitude all show significant negative trends (*P* < 0.05), means that the higher the altitude and temperature caused less precipitation. But the previous study showed that the temperature decreases with the increase in altitude, and the temperature decreases 0.65℃ for every 100 m increase in altitude^35^, this can explain why the coefficient of influence of altitude on rainfall is lower than that of temperature and longitude. Therefore, altitude is not the main influencing factor that causes the spatial unevenness of precipitation in Sichuan Province, the precipitation is determined by multiple factors of geographical features. According to the regression coefficient, annual rainfall decreases by 15.1–25.8 mm for every 100 m of elevation increase, and annual rainfall decreases by 20.7–71.5 mm for every degree of temperature rise, but the largest effect is the change in latitude and longitude, the more eastern part of the Sichuan Province receives less annual rainfall.

**Table 4:** Regression equation of geographical factors (altitude (*H*), temperature (*T*), longitude (*L*_*o*_) and latitude (*L*_*a*_)) and annual precipitation of meteorological stations in different periods.

Period	Regression equation	R^2^	*P*(*H*)	*P*(*T*)	*P*(*L*_*o*_)	*P*(*L*_*a*_)
1961–1970	*Y* = − 0.258 × *H* − 71.5 × *T* − 84.9 × *L*_*o*_ + 51.3 × *L*_*a*_ − 2678.7	0.732	0.025	0.003	0.000	0.011
1971–1980	*Y* = − 0.247 × *H* − 60.7 × *T* − 93.3 × *L*_*o*_ + 67.1 × *L*_*a*_ − 2032.7	0.805	0.020	0.001	0.000	0.014
1981–1990	*Y* = − 0.228 × *H* − 55.0 × *T* − 87.6 × *L*_*o*_ + 70.8 × *L*_*a*_ − 2646.3	0.826	0.034	0.004	0.000	0.011
1991–2000	*Y* = − 0.172 × *H* − 49.6 × *T* − 96.2 × *L*_*o*_ + 61.6 × *L*_*a*_ − 1648.2	0.812	0.050	0.002	0.000	0.007
2001–2010	*Y* = − 0.151 × *H* − 20.7 × *T* − 79.0 × *L*_*o*_ + 105.6 × *L*_*a*_ − 2352.4	0.817	0.050	0.004	0.000	0.000
2011–2019	*Y* = − 0.175 × *H* − 36.6 × *T* − 98.8 × *L*_*o*_ + 73.6 × *L*_*a*_ − 2445.2	0.702	0.043	0.007	0.001	0.000

Regarding *P*(*H*), *P*(*T*), *P*(*L*_*o*_) and *P*(*L*_*a*_) means the result of t test, less than 0.05 indicates that the variable is significant.

Calculate the trend changes of SPEI of different geomorphological type on different time scales according to MK test, we found that the four types all showed negative trends from 1961 to 2019, and the hills and plains regions showed extreme significant negative trends (*P* < 0.01), the trend coefficient of hills and plains is also greater than that of plateaus and mountainous regions, means that the degree of drought in the eastern part of Sichuan Province is gradually aggravated. At the same time, the drought precipitation trend demonstrated decreasing trends transfer from northwest to east and gather in central Sichuan Province. Some previous studies on Southwest China have shown that 65% of the regional droughts in parts of Southwest China have increased significantly in drought intensity and duration, especially in Sichuan Province, and extreme drought indicate that the trend coefficient from east to west in the southwest region is decreasing^14,15^. Therefore, it is particularly important to study the distribution of drought characteristics in Sichuan Province.

Although the degree of drought in the eastern part of Sichuan Province continues to increase, but according to the spatial distribution trends of drought intensity and drought frequency in different periods from 1961 to 2019, we found that the western plateau area of Sichuan Province is still a drought-prone area, and the drought meteorological hotspots are mainly in the plateau and intermountain basins. Wu based on the principle of vegetation water supply index (VWSI) test the Sichuan drought spatial distribution and in-site time series analysis, the results show that drought meteorological hotspots are mainly concentrated in the Sichuan basin, because this area is surrounded by mountains, which is difficult for the continental monsoon to release after the basin sinks, causing this area to become a hotspot with frequent droughts^19^. According to the distribution characteristics of drought in different time and space, it can be helpful to build a targeted drought control strategy, such as strengthening the construction of farmland water conservancy infrastructure regionally, popularizing farmland water-saving technology locality, and adjusting agricultural planting structure periodically.

## Conclusions

In this study, the meteorological and geographical drought characteristic in Sichuan Province of China were evaluated with SPEI, SPI, drought precipitation trend, drought intensity and drought intensity respectively on different time and space scales. The main conclusions are listed as follows:

1. There are significant differences in drought characteristics between SPI and SPEI in some regions and time periods, We used conditional probability (C*p*) to analyze the difference between SPI and SPEI, the C*p* (SPI) decreases continuously while the C*p* (SPEI) increases continuously in the plateau region from 1961 to 2019, and C*p* (SPEI) was greater than C*p* (SPI) on the time scale of all regions indicated that SPEI was more sensitive to drought assessment than SPI, and could more accurately reflect dry/wet alternations in more complex regions.
2. The SPEI drought trend in plain and hilly regions was greater than that in plateau and mountain regions on all time scales (− 0.039 year^−1^ for 1-month in hilly, − 0.035 year^−1^ for 1-month in plain, − 0.14 year^−1^ for 1-month in plateau, − 0.026 year^−1^ for 1-month in mountain), and 80% of the stations in the eastern region showed a negative growth, the magnitude of trend varied between − 4.4 to 0.1 year^−1^ in eastern region, means that the degree of drought in the eastern part of Sichuan Province is gradually aggravated and the drought trends transfer from northwest to east.
3. Based on drought intensity and drought frequency, we found the drought intensity in the eastern part of Sichuan gradually weakened (0.36–0.51), while the drought intensity in the western region gradually increased (0.54–1.05) and drought events mainly occurred in the southwest plateau and central mountainous regions (24–47 times), means that drought meteorological hotspots was mainly concentrated in the Sichuan basin.
4. By using multivariable linear regression method (MLR) to establish a multiple regression model of average annual precipitation in different periods, the correlation coefficient of influence of altitude on rainfall is lower than that of temperature and longitude, indicated altitude is not the main influencing factor that causes the spatial unevenness of precipitation in Sichuan Province. According to the correlation coefficient of each factor, the difference of geographical location and temperature is the main influencing factor of the uneven rainfall.
5. The main reasons for the frequent drought in Sichuan province can be attributed to the synoptic climatology, the uneven spatial and temporal distribution of rainfall and the anomaly of atmospheric circulation. The subsidence is prevalent in Sichuan province all the year round, and the surrounding mountains form a special steamer effect, which exacerbates the climatic drought events.
